# Semisynthetic Modification of Tau Protein with Di-Ubiquitin Chains for Aggregation Studies

**DOI:** 10.3390/ijms21124400

**Published:** 2020-06-20

**Authors:** Francesca Munari, Carlo Giorgio Barracchia, Francesca Parolini, Roberto Tira, Luigi Bubacco, Michael Assfalg, Mariapina D’Onofrio

**Affiliations:** 1 Department of Biotechnology, University of Verona, 37128 Verona, Italy; francesca.munari@univr.it (F.M.); carlogiorgio.barracchia@univr.it (C.G.B.); francesca.parolini@univr.it (F.P.); roberto.tira@univr.it (R.T.); michael.assfalg@univr.it (M.A.); 2 Department of Biology, University of Padova, 35121 Padova, Italy; luigi.bubacco@unipd.it

**Keywords:** tau protein, ubiquitination, semisynthesis, disulfide-coupling, polyubiquitin, fibrils, aggregation, neurodegeneration

## Abstract

Ubiquitin, a protein modifier that regulates diverse essential cellular processes, is also a component of the protein inclusions characteristic of many neurodegenerative disorders. In Alzheimer’s disease, the microtubule associated tau protein accumulates within damaged neurons in the form of cross-beta structured filaments. Both mono- and polyubiquitin were found linked to several lysine residues belonging to the region of tau protein that forms the structured core of the filaments. Thus, besides priming the substrate protein for proteasomal degradation, ubiquitin could also contribute to the assembly and stabilization of tau protein filaments. To advance our understanding of the impact of ubiquitination on tau protein aggregation and function, we applied disulfide-coupling chemistry to modify tau protein at position 353 with Lys48- or Lys63-linked di-ubiquitin, two representative polyubiquitin chains that differ in topology and structure. Aggregation kinetics experiments performed on these conjugates reveal that di-ubiquitination retards filament formation and perturbs the fibril elongation rate more than mono-ubiquitination. We further show that di-ubiquitination modulates tau-mediated microtubule assembly. The effects on tau protein aggregation and microtubule polymerization are essentially independent from polyubiquitin chain topology. Altogether, our findings provide novel insight into the consequences of ubiquitination on the functional activity and disease-related behavior of tau protein.

## 1. Introduction

Ubiquitination is a prevalent post-translational process that regulates essential eukaryotic cell functions such as protein homeostasis, signaling, and cellular localization [1,2,3]. It consists of the covalent addition of the small protein ubiquitin to the side chain of lysine residues in a target protein. The reaction is controlled by the coordinated action of a ubiquitin-activating enzyme (E1), a ubiquitin-conjugating enzyme (E2) and a ubiquitin ligase (E3), which catalyze the formation of an isopeptide bond between the carboxyl group of the C-terminal glycine of ubiquitin and the ε-amino group of the substrate’s lysine [2,3,4,5]. Ubiquitination of a ubiquitin molecule through one (or more) of its seven lysine residues (Lys6, Lys11, Lys27, Lys29, Lys33, Lys48 and Lys63) or its amino terminus produces polymers named polyubiquitin chains [2,5]. Polyubiquitin chains have different biological functions depending on the type of linkage that connects the ubiquitin units. Lys48- and Lys11-linked chains regulate proteasome-mediated protein degradation, Lys63-linked chains are involved in endocytosis, cell signaling, and cellular response to DNA damage [2,6], while the functions of the remaining polyubiquitin chain types remain less clear [7]. Each chain type exhibits unique conformational properties that correlate with the diverse biological activities [7,8]. Prototypical polyubiquitin chains with Lys48- and Lys63- linkage display distinct structures: prevalently compact in the case of Lys48-linked chain [9] and extended in the case of Lys63-linked polyubiquitin [10]. Differences in polyubiquitin chain topology generate unique molecular interfaces that govern the selective recognition of cellular receptors and the fine modulation of specific cellular pathways [11]. 

Due to the central role of protein ubiquitination in fundamental cellular pathways, the dysfunction of the ubiquitin system is involved in the onset of many human pathologies [12]. In particular, the impairment of protein turnover mediated by the ubiquitin proteasome system (UPS) is implicated in neurodegenerative disorders, including Alzheimer’s disease (AD), characterized by the accumulation of misfolded proteins [4]. The understanding of the molecular mechanisms of UPS malfunction in relation to protein aggregation has been the target of intense and recent research aimed at finding new therapeutic strategies for the treatment of these diseases. The major incidence of neurodegeneration in the elderly can be justified in part by the fact that a progressive worsening of clearance activity in aging brains may promote the accumulation of toxic and misfolded proteins within neurons [13,14]. Additionally, it is well established that ubiquitin is a key component of the intracellular deposits formed by misfolded proteins in damaged neurons [4,15] and that certain types of protein aggregates can directly inhibit or obstruct the proteasome machinery [12,16,17]. The connection between UPS and protein aggregation was extensively studied in the case of AD, a progressive brain degeneration that is still incurable and which has a major incidence worldwide. 

**Figure 1:**
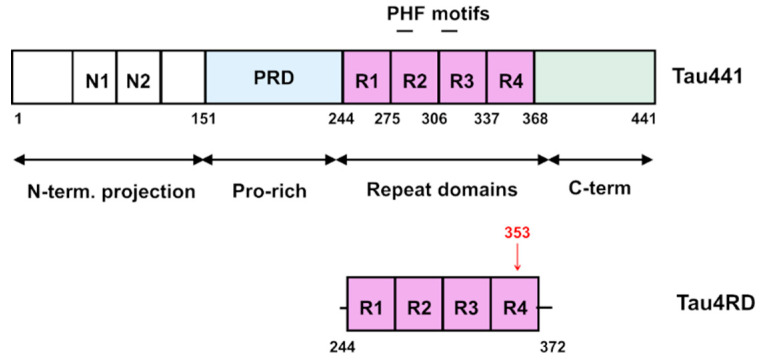
Schematic representation of Tau441 and Tau4RD proteins with their domain organization. The position of residue 353, that is, the conjugation site with di-ubiquitin molecules used in this work, is highlighted in red.

One of the key players in AD pathogenesis is the microtubule associated protein tau, an axonal protein mainly expressed in the central and peripheral nervous system [18]. In the brain, tau protein occurs in six isoforms of different length, generated by alternative splicing [19,20]. Tau protein belongs to the class of intrinsically disordered proteins. The large conformational flexibility that characterizes disordered proteins and regions allows them to carry out a variety of biological activities via distinct recognition mechanisms unfeasible for rigid proteins [21,22,23]. Tau protein controls the stability and assembly of microtubules, a function that is finely regulated by phosphorylation [18]. The extensively studied, 441-residue isoform (Figure 1) includes an N-terminal half that projects from the microtubule surface and a C-terminal half that promotes the assembly of microtubules. The four pseudo-repeats R1-R4 spanning residues 244-369, together with their flanking regions, constitute the microtubule binding domain (MBD) [19]. The MBD includes two hexapeptide motifs (VQIVYK and VQIINK) that are critical in promoting nucleation of tau aggregates [24]. Under pathological conditions, tau protein becomes hyperphosphorylated, detaches from the microtubules and undergoes a complex aggregation process characterized by a conformational transition to β-sheet rich structures and formation of straight and paired helical filaments (PHFs) that accumulate in neurofibrillary tangles (NFT) [19,20]. PHFs isolated from AD-brain or obtained from recombinant tau protein have been shown to be able to inhibit the proteasome [17]. The solved structure of tau filaments purified from AD-brain revealed residues 306-378 as the ordered fibril core, with the N- and C termini forming the fuzzy coat [25]. 

In addition to being heavily phosphorylated, tau protein isolated from AD-PHFs was found to be ubiquitinated at several lysine sites within the MBD [26,27,28]. Specifically, mono-ubiquitin was found to be linked to Lys254, Lys257, Lys311 and Lys317 [27], while polyubiquitin chains were found to be conjugated to Lys254, Lys311 and Lys353 [28]. PHF-tau is modified by three polyubiquitin linkages, Lys6-, Lys11-, and Lys48-, with the latter one being the most prevalent [28]. Recent cryo-EM studies revealed that, besides a proteasome-targeting role, ubiquitination of tau filaments could be structurally involved in mediating specific inter-protofilament packing [26]. Thus, it is plausible that ubiquitination severely affects the assembly and stability of tau filaments and the understanding of the underlying mechanism has the potential to shed new light into the molecular basis of the disease. 

To study the impact of ubiquitination on the mechanism of tau protein aggregation, detailed biophysical experiments necessitate highly homogenous and uniquely modified protein samples. However, homogenous ubiquitination of lysine side chains is rarely achieved with the use of ubiquitin ligase enzymes. Indeed, we recently showed that CHIP (Carboxy terminus of Hsp70-interacting protein), an E3 ligase of tau protein [29], ubiquitinates the protein at more than ten sites [30]. To overcome the inherent limitation of the enzymatic approach and to obtain site-specific ubiquitination of target proteins, chemists have developed a vast array of semisynthetic methods that are based on the chemical conjugation of protein precursors [31]. Chemical ubiquitination strategies based on non-native isopeptide bond formation are often easier to implement and give high yields of the product conjugates. Among these, disulfide-coupling chemistry has proven to be highly versatile, efficient and robust, and has already been applied in many studies [30,31,32,33,34]. As a replacement for isopeptide linkage, this semisynthetic method generates a disulfide bond between a Cys residue placed in a specific position of the target protein and the C-terminal aminoethanethiol of a ubiquitin derivative obtained by intein processing. Recently, we described the production and characterization of tau protein mono-ubiquitinated at three different positions using disulfide-directed methodology [30]. With this approach, we obtained novel insight into the diverse effects that lysine mono-ubiquitination at different sites exerts on tau protein aggregation. 

Here, we introduce a method based on directed disulfide bond formation in combination with enzymatic synthesis of ubiquitin chains to obtain controlled polyubiquitination of tau protein, thus adding a further level of complexity in the study of ubiquitinated tau protein. We produced and characterized the aggregation process of tau protein species homogenously modified with di-ubiquitin molecules at position 353, one of the ubiquitinated sites found in AD-brain filaments. The versatility of the method allowed us to incorporate two types of di-ubiquitin chains (Lys48- or Lys63- chains) characterized by different structures, and to explore the impact of these prototypical ubiquitin polymers on tau protein aggregation.

## 2. Results

In the present work, we optimized a semisynthetic strategy based on disulfide-coupling chemistry to covalently attach a di-ubiquitin molecule to the tau protein. Ubiquitin-substrate conjugates obtained using this method are functional mimics of the native ubiquitinated counterparts, with the synthetic linkage being only one atom longer than the native isopeptide bond [32,34]. We selected the construct tau4RD, which represents a short form of the protein, spanning residues Q244-E372 plus an initial Met (Figure 1). Tau4RD has been widely used to study the aggregation behavior of tau protein as it includes the majority of the microtubule binding region and most of the residues involved in the assembly of the cross-β structure forming the core of the tau filaments [35]. To obtain site-specificity, we used a tau4RD mutant devoid of the endogenous cysteines and where a single cysteine was installed at the desired position. We chose to introduce the modification at position 353 because this site was found polyubiquitinated in PHF-tau [26,28], it is part of the fibril core found in different tauopathies [26], and mono-ubiquitination at this position had an observable influence on the aggregation kinetics without hindering the formation of short fibrils [30]. Thus, it was deemed suitable for evaluating the effect of an extended ubiquitin chain on tau filament assembly.

**Figure 2:**
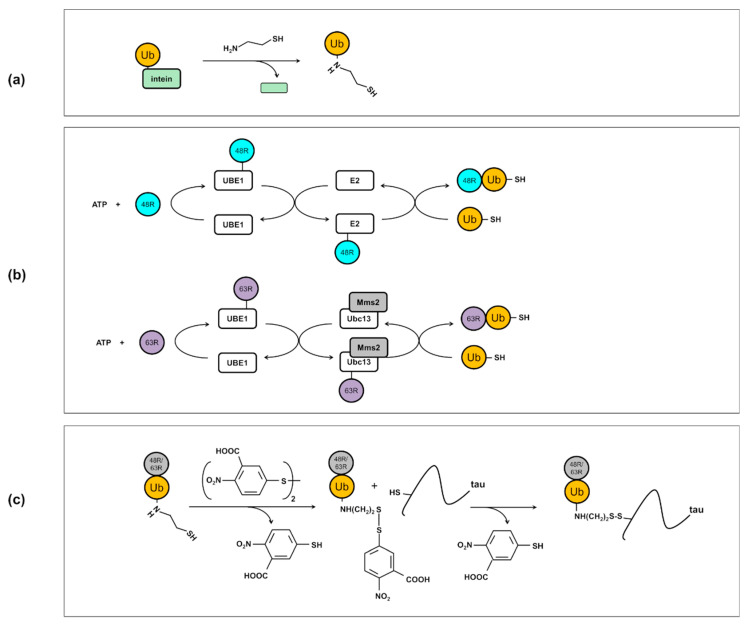
(**a**) Scheme of cysteamine-mediated cleavage of Ub-SH from a ubiquitin-intein fusion protein. We produced a chimeric protein where ubiquitin was cloned to the N-terminal of the GyrA intein. A ubiquitin with a C-terminal aminoethanethiol linker (Ub-SH) was obtained through a trans-thioesterification reaction between intein and cysteamine, followed by a S-to-N acyl shift. (**b**) Scheme of production of Ub_2_(48)-SH and Ub_2_(63)-SH di-ubiquitin molecules by enzymatic reaction. For the controlled assembly of K48-linked chains, the enzymes E1 and E2-25K were used. For the K63-linked chains, we used E1 and the complex Mms2/Ubc13. (**c**) Reaction of Ub_2_(48/63)-SH with 5,5′-Dithiobis(2-nitrobenzoic acid) gave an activated asymmetric disulfide. This was then allowed to react with the unique cysteine placed in position 353 in tau4RD to obtain the disulfide-linked Ub_2_(48)tau4RD(353) and Ub_2_(63)tau4RD(353) conjugates.

**Figure 3:**
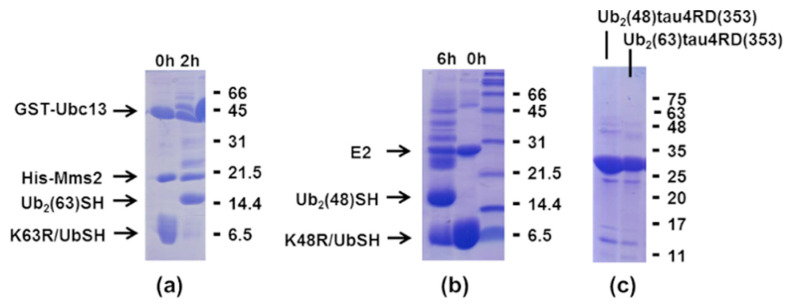
SDS-PAGE showing the enzymatic reaction to obtain the Ub_2_(63)-SH (**a**) or Ub_2_(48)-SH (**b**) di-ubiquitin molecules. In (**c**), the purified Ub_2_(48)tau4RD(353) and Ub_2_(63)tau4RD(353) are shown.

**Figure 4:**
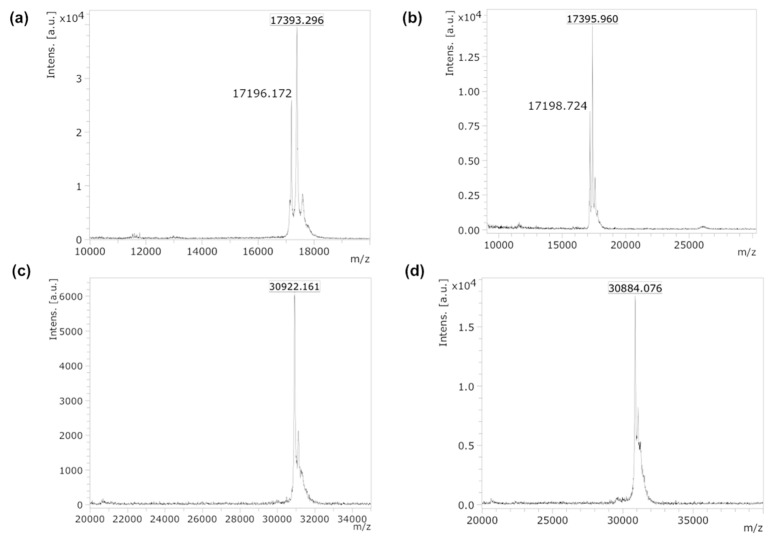
Maldi TOF MS analysis of protein samples. We obtained mass values of 17,393 and 17,396 for the Ub_2_(48)-S-TNB (**a**) and Ub_2_(63)-S-TNB (**b**) adducts, respectively (expected mass 17397), where TNB stands for (2-nitro-5-thiobenzoic acid). In (**a**) and (**b**), the MS peak of the unconjugated Ub_2_(48/63)SH protein is also present (expected mass 17200). We obtained mass values of 30,922 and 30,884 for the Ub_2_(48)tau4RD(353) (**c**) and Ub_2_(63)tau4RD(353) (**d**), respectively (expected mass 30923).

The method of preparation of di-ubiquitinated tau4RD is illustrated in Figure 2. First, using linkage-specific enzymes, we assembled di-ubiquitin molecules with Lys48- and Lys63-linkage (Figure 2b and Figure 3a,b). We used the ubiquitin mutants Lys48Arg or Lys63Arg as distal units and Ub-SH, a ubiquitin derivative bearing a C-terminal aminoethanethiol linker obtained from intein cleavage with cysteamine, as proximal unit (Figure 2a). Homogenous di-ubiquitin chains ending with a C-terminal thiol (Ub_2_(48/63)-SH) were then purified and allowed to react with 5,5′-Dithiobis(2-nitrobenzoic acid) (DTNB) to produce asymmetric activated disulfides (Figure 2c and Figure 4a,b). Finally, the activated disulfides were incubated with tau4RD, modified with a unique cysteine at position 353, to produce the disulfide-linked conjugates Ub_2_(48)tau4RD(353) and Ub_2_(63)tau4RD(353) (Figure 2c). The protein conjugates were obtained at purity of >95% (Figure 3c) and their identity was verified by mass spectrometry (Figure 4c,d). These samples were then used to perform aggregation experiments and their behavior was compared with that of mono-ubiquitinated Ub-tau4RD(353) and of the unconjugated cysteine-free protein (tau4RD∆C). 

**Figure 5:**
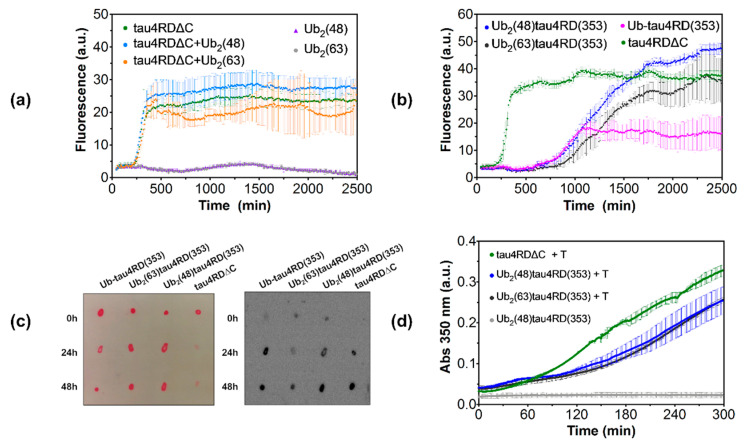
(**a**,**b**) Thioflavin T-based aggregation kinetics experiments. Error bars of fluorescence curves correspond to standard deviations of at least three independent experiments. In (**a**), aggregation data of tau4RD∆C are reported, in the absence or presence of equimolar Ub_2_(48/63). Kinetics data for the Ub_2_(48/63) alone are also reported. For these control experiments Ub_2_(48/63) were produced with a distal D77 ubiquitin mutant, to avoid insertion of free thiol groups. In (**b**), aggregation data of di-ubiquitinated forms Ub_2_(48)tau4RD(353) and Ub_2_(63)tau4RD(353) are reported in comparison with tau4RD∆C, and mono-ubiquitinated Ub-tau4RD(353). (**c**) On the right, dot Blot of Ub_2_(48)tau4RD(353) and Ub_2_(63)tau4RD(353) at different times of aggregation induced with equimolar heparin, probed with the A11 antibody. On the left, deposited proteins were stained with Ponceau. Samples of tau4RD∆C and Ub-tau4RD(353) were used as controls. (**d**) Microtubule assembly in the presence of tau protein samples was measured monitoring the absorbance at 350 nm. Error bars of absorbance curves correspond to standard deviations of three independent experiments. Ub_2_(48)tau4RD(353) sample in the absence of tubulin was used as control.

The kinetics of filament formation of the prepared proteins was followed by monitoring changes in Thioflavin T (ThT) fluorescence (Figure 5a,b). In this assay, the fluorescence emission of ThT increases upon its specific binding to the β-sheet rich structure characteristic of tau filaments. The sigmoidal profile of the kinetic curves reflects the cooperative nature of the nucleation-dependent aggregation process. The initial flat portion of the curve, corresponding to the lag phase, is followed by a steep transition (the growth or elongation phase) and a flat terminal part (plateau phase). As shown previously [30], aggregation of tau4RD∆C in the presence of heparin is very rapid, characterized by an early transition midpoint at ~5 h, and a fast fibril growth, with an elongation time of 0.7 ± 0.2 h (Figure 5a,b, Table 1). The addition of unconjugated di-ubiquitin molecules (Ub_2_(48/63)) at an equimolar ratio with tau4RD∆C did not affect the aggregation curves (Figure 5a), excluding the possibility that significant tau-ubiquitin inter-molecular contacts interfered with filament formation. Moreover, in the experimental conditions used, neither Lys48- nor Lys63-linked di-ubiquitin formed fibrils by themselves (Figure 5a), thereby allowing us to interpret the experimental data in terms of ubiquitination-induced perturbations of the aggregation of tau protein. 

The sigmoidal aggregation profiles obtained with Ub_2_(48)tau4RD(353) and Ub_2_(63)tau4RD(353) indicate that di-ubiquitinated tau protein samples were capable of forming filamentous aggregates (Figure 5b). From visual inspection of the aggregation curves measured on all of the tau protein samples, we noted a significant difference in the maximum fluorescence at plateau for the mono-ubiquitinated tau protein in comparison with the other proteins. Because signal intensity is influenced by specific amyloid properties and the proteins under investigation have different structures and shapes, the reduction in ThT fluorescence observed for mono-ubiquitinated tau protein was likely due to a different affinity for ThT and not to the number of fibrils formed.

Based on a quantitative analysis of the aggregation kinetics, we found that filaments formation by di-ubiquitinated tau protein was significantly delayed compared to the unconjugated protein, as deduced by the longer lag phase and elongation time of the di-ubiquitinated proteins (Figure 5b, Table 1). Additionally, di-ubiquitination was found to specifically affect the transition midpoint and elongation time more than mono-ubiquitination. Indeed, Ub_2_(48)tau4RD(353) and Ub_2_(63)tau4RD(353) displayed a similar transition half time of ~21 h, which was significantly shifted with respect to the value determined for Ub-tau4RD(353) (t_0.5_ ~15 h), and all the values of modified tau protein were larger compared to that of tau4RD ΔC (t_0.5_ ~5 h). Likewise, the elongation time showed an analogous trend for the investigated samples (Table 1). By comparison of the aggregation curves, it emerged that the kinetics of the two di-ubiquitinated proteins was similar.

**Figure 6:**
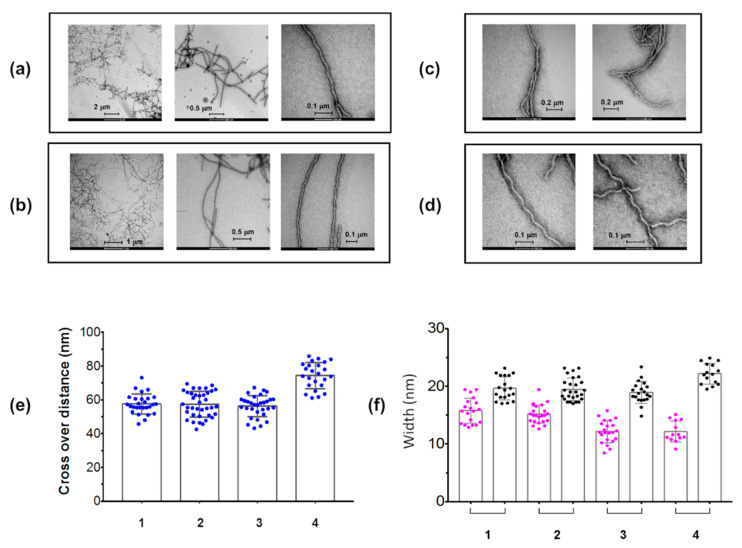
TEM representative images of (**a**) Ub_2_(48)tau4RD(353), (**b**) Ub_2_(63)tau4RD(353), (**c**) tau4RDΔC, and (**d**) Ub-tau4RD(353), after 48 h of incubation at 37 °C under static condition. 30 µL of sample at a concentration of 2.5 µM were deposited. Distributions of (**e**) cross-over distances and (**f**) widths of the twisted filaments measured from TEM images of Ub_2_(48)tau4RD(353) (1), Ub_2_(63)tau4RD(353) (2) and Ub-tau4RD(353) (3) conjugates and tau4RDΔC (4). In (**e**) and (**f**), dots indicate single measurements and bars the positions of means ± SD. In (**f**), dots in magenta refer to the measures of narrow widths and in black to large widths.

Ub_2_(48)tau4RD(353) and, to a lesser extent Ub_2_(63)tau4RD(353), produced aggregates that reacted with A11 (Figure 5c), an antibody capable of recognizing prefibrillar oligomers of diverse proteins [36]. Thus, the ability of the tau protein component to form intermediate amyloidogenic species was maintained after modification, as shown previously for mono-ubiquitinated tau4RD [30]. The ability of both Ub_2_(48)tau4RD(353) and Ub_2_(63)tau4RD(353) to form mature fibrils was confirmed by the TEM images (Figure 6a,b), which showed the presence of well-formed twisted filaments. The morphological analysis revealed that filaments were characterized by a large width of 20 ± 2 nm and a narrow width of 15–16 nm, and a twist crossover repeat of 57-58 nm (Table 2), indicating that the overall morphology of tau4RDΔC filaments was not heavily modified by di- or mono-ubiquitin conjugation (Figure 6a–d, Table 2). 

Taken together, the obtained data clearly indicate that the incorporation of protein modifiers in the microtubule binding domain of tau protein at position 353 interferes with the aggregation mechanism but does not abrogate the formation of mature fibrils. The addition of one ubiquitin moiety to tau protein determines the inhibition of fibrils formation. The inhibitory effect is even stronger when a second ubiquitin moiety is attached to the proximal ubiquitin unit, as it resulted from the substantial increase of the duration of both midpoint transition and elongation time for the di-ubiquitinated species. However, despite their known structural differences, the topology of the investigated di-ubiquitin molecules (Lys48- or Lys63-linked) did not influence the process of fibrils formation. Thus, it appears that the inhibitory effect of di-ubiquitination at the 353 site is caused by the increased steric hindrance which impairs microscopic events that lead to fibrils formation. 

After having established how di-ubiquitination affects tau fibril formation, a process associated with disease, we set out to describe its consequence on tau protein functional activity. Specifically, we investigated the impact of Lys48- and Lys63-linked di-ubiquitin on tau-mediated tubulin polymerization. The assay was performed using Ub_2_(48)tau4RD(353) or Ub_2_(63)tau4RD(353), and in the presence of tau4RD∆C as a control (Figure 5d). Microtubule (MT) polymerization was monitored by following the increase in absorbance at 350 nm. The kinetics of MT assembly in the presence of tau4RDΔC resembled previous results reported on tau4RD [37]. The data acquired in the presence of Ub_2_(48)tau4RD(353) or Ub_2_(63)tau4RD(353) indicates that the presence of the di-ubiquitin chains at position 353 of tau protein, moderately but significantly inhibits MT polymerization. Indeed, after 300 min of incubation of tubulin with the conjugates, we observed ~77% of MT formation (referred to 100% for tau4RDΔC). This effect was independent from the topology of the di-ubiquitin chain, as the observed curves were almost superimposable.

## 3. Discussion

The microtubule-associated protein tau plays a central role in the pathogenesis of AD. Besides being heavily phosphorylated [20], tau protein in PHFs from AD brains is found to be mono- or polyubiquitinated at multiple sites [26,27,28]. The observation that most of these sites belong to the microtubule binding region, a domain involved in the formation of the filaments core, suggests the attempt of the neurons to get rid of aggregated tau protein forms via the UPS. This hypothesis is supported by the fact that PHF-tau was found to be modified by Lys48- and Lys11- linked chains [28], both being signals for proteasomal degradation, and by mono-ubiquitin, recently recognized as an additional signal for proteasomal targeting [38]. These observations suggest a possible role of ubiquitination in the formation and clearance of pathological tau protein species.

In a bid to advance our understanding of the impact of ubiquitination on tau protein aggregation and function, we recently started developing methods to attain controlled ubiquitination of tau protein samples for molecular-level investigations. In a previous work, we produced mono-ubiquitinated tau4RD samples and found that the impact of the modification on tau protein fibrillogenesis was site-dependent [30]. While ubiquitination at Lys311, located within the PHF motif of the R3 domain, essentially abolished filament formation, modification at Lys254 and Lys353 changed the aggregation kinetics but did not arrest fibrillation completely. To follow up on this study, in the present work, we set out to investigate whether a minimal polyubiquitin chain, a di-ubiquitin, could affect the aggregation behavior of tau protein.

To obtain site-specific di-ubiquitination, we combined enzyme-mediated preparation of di-ubiquitin derivatives with disulfide-directed ligation to tau4RD, expanding our approach originally developed to produce mono-ubiquitination [30]. Based on the previous findings with mono-ubiquitination, di-ubiquitin was installed at position 353, a site that was deemed suitable for evaluating the effect of an extended ubiquitin chain on filament assembly. Because polyubiquitin chains exist in a variety of topologies and structures, here we chose to investigate the prototypical Lys48- and Lys63-linked chains as being representative of polymers that preferentially adopt compact or extended conformations, respectively [7]. Lys48-linked di-ubiquitin predominantly adopts a closed conformation in which the functional hydrophobic patches of mono-ubiquitin are sequestered at the ubiquitin/ubiquitin interface [9]. In contrast, Lys63-linked di-ubiquitin chains are extended and adopt an open conformation with no direct contact between the neighboring ubiquitin subunits, thereby exposing the hydrophobic patches of mono-ubiquitin [10]. Thus, we hypothesized that different architectures of polyubiquitin chains could differently affect substrate aggregation.

The performed aggregation experiments revealed that both di-ubiquitinated tau protein conjugates were less prone to form fibrils compared to the unconjugated protein (Figure 5b), although they retained the capability to form A11-positive prefibrillar oligomeric species (Figure 5c) and twisted filaments (Figure 6). Moreover, the dimeric ubiquitin modifier increased significantly both the half-time of the transition and the fibril elongation time with respect to mono-ubiquitin, with the time constants following the order: di-ubiquitinated >> mono-ubiquitinated > unmodified (Figure 5b, Table 1). However, the topological differences between the two di-ubiquitin chains (Lys48- or Lys63-linked) tested do not differentially interfere with the process of fibrils formation. 

It is established that the duration of the lag phase depends on the rates of multiple parallel microscopic processes, such as the formation of primary nuclei and their amplification through elongation and secondary nucleation processes [39]. Because variations of the former process do not affect the growth phase, while changes in elongation and secondary nucleation modify both the lag phase and the growth phase, it seems possible that di-ubiquitination of tau protein could interfere with the growth and proliferation of primary nuclei rather than with their formation. This was more evident for di-ubiquitinated, rather than mono-ubiquitinated, tau protein. Since the inhibitory effect was dependent on the length of the ubiquitin chain, the impact on tau protein aggregation is likely be a result of a combination of structural motif at the tau-ubiquitin conjugating point and of the presence of the distal ubiquitin moiety which adds steric hindrance. Yet, we do not exclude the possibility that a further elongation of the polyubiquitin chain could elicit different results, particularly if considering that longer ubiquitin chains have lower thermodynamic stability and can form fibrils themselves under specific conditions [40]. 

To interpret our results in the wider context of post-translational modifications of tau protein, we reviewed recent studies which explored the impact of single or multiple phosphorylation of full-length tau protein or tau4RD [37,41]. Phosphorylation of both Ser262 and Ser356 was found to significantly alter the aggregation kinetics of tau4RD [37], and single-site phosphorylation affected tau protein aggregation in a sequence-specific manner [37,41]: phosphorylation of Ser305 had a significant impact on fibril formation; however modification at Ser356 did not significantly perturb the aggregation of tau protein. Here we have shown that di-ubiquitination at 353, close to residue 356, allows the formation of fibrils, although with a reduced aggregation rate, in line with the observation that this site is excluded from the ordered core (residues 272–330) in heparin-induced tau filaments [42]. Additionally, recent cryo-EM studies show that tau filaments from brain tissues [25,26] are characterized by a long core that includes Lys353 and suggest that bulky modifiers, such as mono- or di-ubiquitin, in that position could affect the mechanism of formation of tau protein aggregates. 

Tau protein is known to play a central role in the assembly and stabilization of microtubules [18]. Therefore, we explored whether di-ubiquitination could regulate tau-mediated tubulin polymerization. Based on our observations, it emerges that the presence of either Lys48- or Lys63-linked di-ubiquitin at position 353 moderately but significantly inhibits MT polymerization. This effect was found to be independent of the di-ubiquitin linkage topology (Figure 5d). Our findings are consistent with previous studies reporting that multiple mono-ubiquitination of tau protein reduces MT binding affinity [43], and phosphorylation at Ser356 (close to Lys353) slightly impairs MT assembly [37]. Taken together, the evidence on site-specific phosphorylated or on multi mono-ubiquitinated tau protein, and our results obtained with the di-ubiquitinated proteins indicate that post-translational modifications around position 353 do not crucially affect tau-assisted MT polymerization and that the functional activity of tau protein is highly dependent on the sites of modification. 

In conclusion, we were able to obtain, for the first time, samples of tau protein conjugated to Lys48- or Lys63- linked di-ubiquitin at a specific site and to study the effect of these modifications on tau protein aggregation and function in comparison with the mono-ubiquitinated species. We demonstrated that the conjugation of tau protein to di-ubiquitin chains at the 353 position significantly delayed, but did not completely inhibit, tau protein aggregation, in analogy with mono-ubiquitination. Quantitative analysis of the aggregation kinetics revealed a more pronounced effect of di-ubiquitin compared to mono-ubiquitin during elongation of filaments, rather than during primary nucleation events. However, linkage topology had a minor effect on the measured kinetic parameters. Finally, we demonstrated that di-ubiquitination modulated the tau-mediated microtubule assembly, thus highlighting the potential role of ubiquitination in the regulation of tau protein function. For a more comprehensive overview, further efforts will have to be directed to produce tau protein modified with longer polyubiquitin chains linked in different positions. 

We believe that our findings also provide crucial information in light of recent structural studies on tau filaments from degenerated brain tissues. Ubiquitin is proposed to play a structural role by mediating specific inter-protofilament packing and promoting the formation of fibril subtypes, specific of the different tauopathies [26]. Moreover, future investigations are required to elucidate the synergic effects played by different post-translational modifications, such as ubiquitination, phosphorylation and acetylation, in the transition of tau protein to toxic species, with the aim to understand their implications in the different pathologies. 

## 4. Materials and Methods 

### 4.1. Materials 

Cysteamine, Tris(2-carboxyethyl)phosphine, 5,5′-Dithiobis(2-nitrobenzoic acid), Thioflavin T, Heparin (H3393), dithiothreitol (DTT), acetonitrile, and Trifluoroacetic acid (TFA) were purchased from Sigma Aldrich (St Louis, MO, USA); Tubulin (T240) was purchased from Cytoskeleton (Denver, CO, USA), italian distributor Società italiana chimici (Rome, IT).

### 4.2. Methods

#### 4.2.1. Protein Expression and Purification

In this work we used the following tau4RD (Q244-E372 plus initial Met) protein variants: tau4RD C291A, C322A (here named tau4RD∆C) and tau4RD C291A, C322A, K353C, here named tau4RD(353). The two proteins were expressed without affinity tag and purified as described in [30]. 

The ubiquitin mutants K48R, K63R, D77 were produced with the same protocol used for wild-type ubiquitin that is described in [44].

The recombinant enzymes human His-tagged E1, GST-tagged E2–25K, yeast His-tagged Mms2, yeast GST-tagged Ubc13 were produced as previously described [45]. In the present work, the GST tag was removed from the E2–25K protein by incubating the clean fusion protein attached to the GSH-resin with thrombin.

To obtain a ubiquitin bearing an aminoethanethiol C-terminal group (Ub-SH) required for the disulfide-coupling reaction, we first produced a chimeric protein where ubiquitin was cloned to the N-terminal of the GyrA intein in a pET22 vector. In this way, the chimeric protein has a C-terminal His-tag. The Ub-intein-His protein was produced in BL21(DE3) cells at 37 °C overnight in auto-inducing medium and purified by immobilized nickel affinity chromatography (IMAC) according to standard protocols. Cleavage of Ub-SH was obtained by incubating the clean fusion protein in a buffer at pH 7.5 containing: Tris-HCl 20 mM, EDTA 1 mM, cysteamine 40 mM, and Tris(2-carboxyethyl)phosphine (TCEP) 3 mM for 48 h at 10 °C. Ub-SH was further purified by reverse -IMAC and a superdex-75 gel filtration column when required. 

#### 4.2.2. Production of Di-Ubiquitin Chains

Di-ubiquitin chains (Ub_2_) were obtained from Ub variants through enzymatic reactions, with the strategy described in [46,47]. The reaction buffer contained 50 mM Tris HCl pH 8.0, 5 mM MgCl_2_, 10 mM ATP, 3 mM TCEP, 0.02% NaN_3_ and protease inhibitors. The ubiquitin variants K48R+D77 and K48R+UbSH were used as building blocks for the reconstitution of Ub_2_(48) and Ub_2_(48)-SH, respectively. For the assembly of these K48-linked chains, we used 1 μM E1 and 20 μM E2-25K. The ubiquitin variants K63R+D77 and K63R+UbSH were used to assemble Ub_2_(63) and Ub_2_(63)-SH, respectively. For these K63-linked chains, we used 1 μM E1, 25 μM Ubc13 and 25 μM Mms2. After reaction, we incubated the solutions with 0.2% perchloric acid to obtain precipitation of most of the enzymes. Then, SP cation-exchange and superdex-75 gel filtration were performed to obtain the pure di-ubiquitin chains. 

#### 4.2.3. Disulfide-Coupling Reaction

First, Ub_2_(48/63)-SH chains were activated with 5,5′-Dithiobis(2-nitrobenzoic acid) (DTNB). Typically, 4-6 mg of DTNB were dissolved in 3 mL of 100 mM Hepes buffer pH 7.0 containing Ub_2_-SH at a concentration of 1-2 mg/mL. After incubation at 10 °C overnight, the obtained Ub_2_(48/63)-S-(2-nitro-5-thiobenzoic acid) (hereafter Ub_2_(48/63)-S-TNB) disulfide adducts were purified by desalting. The activated proteins were verified by MALDI (Figure 4a,b). 

Then, the Ub_2_(48/63)-S-TNB disulfide adducts were incubated with tau4RD(353) in equimolar amount in 100 mM Hepes buffer pH 7.0, for 20’ at 25 °C. The Ub_2_(48/63)tau4RD(353) disulfide conjugates were then purified by SP-ion exchange chromatography and verified by MALDI (Figure 4c,d). 

It is important to note the thiol containing proteins Ub_2_(48/63)-SH and tau4RD(353) were first incubated with a large excess of DTT that was then properly removed by size exclusion chromatography just before the disulfide coupling reaction.

The procedure to obtain mono-ubiquitinated tau protein at position 353 (Ub-tau4RD(353)) by a similar disulfide-coupling strategy is described in [30].

#### 4.2.4. Thioflavin T Aggregation Assay

Thioflavin T aggregation assays were carried out in 96-well dark plates in a Tecan Infinite M200PRO Plex plate reader at 30 °C with cycles of 30 s of orbital shaking at 140 rpm and 10 min of rest throughout the incubation. ThT fluorescence measurements were taken every 11 minutes, using excitation wavelength of 450 nm and recording fluorescence emission at 480 nm.

Samples contained 0.01 mM proteins in 20 mM sodium phosphate buffer at pH 7.4 and 50 mM NaCl (with 0.02% NaN_3_ and protease inhibitors with EDTA), incubated with equimolar amount of heparin and ThT. Samples containing tau protein variants were filtered through a 100 kDa cut-off filter (Sartorius) to remove pre-existing large oligomers. Each measurement was performed in three replicates. The aggregation curves were analyzed by fitting each individual experimental data set with the following sigmoidal function [48]:

$$y=y_{i}+\frac{y_{f}}{1+e^{-\left[\left(t-t_{0.5}\right)/\tau \right]}}$$

where *y* is the fluorescence intensity as a function of time *t*, *y*_i_ and *y*_f_ are the intercept of the initial and final baselines with the *y*-axis, *t*_0.5_ is the time needed to reach halfway through the elongation phase and τ is the elongation time constant. The lag time is defined as *t*_lag_ = *t*_0.5_ − 2τ. The values reported in the text correspond to the mean ± SD of the individual values computed separately on each curve. 

Analysis and figures production were carried out with GraphPad Prism 7 (GraphPad Software Inc., La Jolla, CA, USA). 

#### 4.2.5. TEM Analysis 

Ub_2_(48)tau4RD(353) sample was further purified by HPLC-reverse phase C18 done with TFA 0.1%/acetonitrile gradient, before buffer exchange to the aggregation buffer.

Samples of Ub_2_(48/63)tau4RD(353) conjugates, tau4RD∆C, and Ub_-_tau4RD(353) were incubated at concentration of 0.05 mM in 20 mM sodium phosphate buffer at pH 7.4 and 50 mM NaCl (with 0.02% NaN_3_ and protease inhibitors with EDTA) at 37 °C without agitation, with the addition of equimolar amount of heparin as aggregation initiator. After 48 h of incubation, we used 100 kDa cut-off filters (Sartorius, Aubagne, FR) to exchange the buffer to H_2_O mQ. 

For TEM measurements, we used a Tecnai G^2^ (FEI, Hillsboro, OR; USA) transmission electron microscope operating at 100 kV, with the procedure described in [30]. Images were analyzed with the ImageJ software.

#### 4.2.6. Dot Blotting

Dot blotting of Ub_2_(48)tau4RD(353) and Ub_2_(63)tau4RD(353) conjugates, tau4RD∆C, and Ub_-_tau4RD(353) at different aggregation times, using the anti-oligomer antibody A11 (ThermoFisher, Waltham, MA, USA), was carried out according to the protocol described in [30]. 

#### 4.2.7. Tubulin Polymerization Assay

The tubulin polymerization assay for tau4RD∆C, Ub_2_(48)tau4RD(353) and Ub_2_(63)tau4RD(353) was initiated by mixing in a 96-well plate 25 μM protein with tubulin (36 μM, in 100 μL total volume) in MT assembly buffer (80 mM PIPES pH 6.9, 2 mM MgCl_2_ and 0.5 mM EGTA) supplemented with 1 mM GTP. The plate was incubated at 37 °C for 4 min. Then, the polymerization was monitored by measuring the absorbance at 350 nm every 30 sec, using a Tecan Infinite M200PRO Plex plate reader (Männendorf, CH) at 37 °C. Each experiment was performed in triplicate.

#### 4.2.8. Mass Spectrometry

Maldi TOF MS analysis was performed on a Bruker Ultraflextreme MALDI-TOF/TOF instrument (Bruker Daltonics, Billerica, MA, USA) by the Centro Piattaforme Tecnologiche of the University of Verona as previously described [30]. 

## Figures and Tables

**Table 1:** Kinetic parameters for the aggregation of tau protein samples, determined on the basis of ThT fluorescence assays.^1^

	t_0.5_ (h)	t_lag_ (h)	τ (h)
tau4RDΔC	5.2 ± 0.1	3.8 ± 0.5	0.7 ± 0.2
Ub-tau4RD(353)	14.8 ± 1.0	12.4 ± 0.1	1.1 ± 0.6
Ub_2_(48)-tau4RD(353)	20.7 ± 1.2	12.1 ± 1.1	4.3 ± 0.1
Ub_2_(63)-tau4RD(353)	21.1 ± 3.3	15.1 ± 1.4	3.0 ± 0.9

^1^ t_0.5_: midpoint of the transition; τ: elongation time constant; t_lag_ = t_0.5_ − 2τ.

**Table 2:** Morphological properties of the twisted filaments of tau protein samples obtained from the analysis of TEM images.

	Large Width	Narrow Width	Crossover Distance
tau4RDΔC	22 ± 2 nm (n = 14)	12 ± 2 nm (n = 13)	74 ± 8 nm (n = 24)
Ub-tau4RD(353)	19 ± 2 nm (n = 20)	12 ± 2 nm (n = 22)	56 ± 6 nm (n = 31)
Ub_2_(48)-tau4RD(353)	20 ± 2 nm (n =19)	16 ± 2 nm (n = 19)	58 ± 6 nm (n = 27)
Ub_2_(63)-tau4RD(353)	20 ± 2 nm (n = 26)	15 ± 2 nm (n = 24)	57 ± 8 nm (n = 37)

## Abbreviations

AD	Alzheimer’s Disease
UPS	Ubiquitin Proteasome System
MBD	Microtubule Binding Domain
PHFs	Paired Helical Filaments
NFT	Neurofibrillary Tangles
DTNB	5,5′-Dithiobis(2-nitrobenzoic acid)
MT	Microtubules
